# Mitigation of drought-induced stress in sunflower (*Helianthus annuus* L.) via foliar application of Jasmonic acid through the augmentation of growth, physiological, and biochemical attributes

**DOI:** 10.1186/s12870-024-05273-4

**Published:** 2024-06-22

**Authors:** Farkhanda Ashraf, Ejaz Hussain Siddiqi

**Affiliations:** Department of Botany, University of Gujrat, Gujrat, 50700 Pakistan

**Keywords:** Biomass production, Gas exchange attributes, Drought stress, Sunflower crop, Jasmonic acid

## Abstract

Drought stress poses a significant threat to agricultural productivity, especially in areas susceptible to water scarcity. Sunflower (*Helianthus annuus* L.) is a widely cultivated oilseed crop with considerable potential globally. Jasmonic acid, a plant growth regulator, plays a crucial role in alleviating the adverse impacts of drought stress on the morphological, biochemical, and physiological characteristics of crops. Experimental detail includes sunflower varieties (Armani Gold, KQS-HSF-1, Parsun, and ESFH-3391), four drought stress levels (0, 25%, 50%, and 75% drought stress), and three levels (0, 40ppm, 80ppm) of jasmonic acid. The 0% drought stress and 0ppm jasmonic acid were considered as control treatments. The experimental design was a completely randomized design with three replicates. Drought stress significantly reduced the growth in all varieties. However, the exogenous application of jasmonic acid at concentrations of 40ppm and 80ppm enhanced growth parameters, shoot and root length (1.93%, 19%), shoot and root fresh weight (18.5%, 25%), chlorophyll content (36%), photosynthetic rate (22%), transpiration rate (40%), WUE (20%), MDA (6.5%), Phenolics (19%), hydrogen peroxide (7%) proline (28%) and glycine betaine (15–30%) under water-stressed conditions, which was closely linked to the increase in stomatal activity stimulated by jasmonic acid. Furthermore, JA 80 ppm was found to be the most appropriate dose to reduce the effect of water stress in all sunflower varieties. It was concluded that the foliar application of JA has the potential to enhance drought tolerance by improving the morphological, biochemical, and physiological of sunflower.

## Introduction

Abiotic stress occurs in any ecosystem when climatic and soil conditions become unfavorable for crop growth and development. Salinity, extreme temperatures, radiation, and drought, among others, are major causes of the degradation of natural ecosystems [1]. Drought is the most severe, as it exacerbates the negative effects of other stresses. Areas prone to drought usually experience less or erratic rainfall due to a disrupted hydrological cycle. Approximately 33% of the world’s agricultural land is vulnerable to drought, which can significantly decrease growth and yield. About one-third of the world’s land area lies in arid and semi-arid regions [2], and according to predictions by 2050, more than 50% of global agricultural land will have to grieve from drought stress. Among water-deficient countries, Pakistan is ranked third in terms of drought-ridden areas and is expected to face severe drought until 2025. The impact of drought stress on plants varies depending on the growth phase, genetic makeup, intensity, and duration of stress [3]. However, it is always challenging for plants from germination to maturity, as it inhibits cell division, reduces shoot length, weakens the root system, leads to biomass reduction, fewer leaves, and lower relative water content [4].

There is stomatal closure [5], decreased stomatal conductance, and reduced transpiration rate to compensate for dehydration. Under such conditions, water use efficiency (WUE) increases at the cellular level [6]. Water deficiency in mesophyll tissues reduces turgor pressure and photosynthetic pigments [7]. Drought enhances the production of hydrogen peroxide (H_2_O_2_), which has oxidative effects on cell membrane integrity, causing lipid peroxidation and the synthesis of malondialdehyde content [8]. Under such circumstances, proline and glycine betaine act as osmoprotectants and help in osmotic adjustment by maintaining turgor pressure [9]. Phenolic content also rises and plays a crucial role as an integral component of the secondary cell wall [10].

Plants utilize various strategies in water-scarce soil, such as leaf rolling, alterations in root and shoot dynamics, and the upregulation of protective substances [11]. Improving germplasm through the exogenous application of growth regulators enhances water use efficiency (WUE) in crops [12]. The exogenous application of growth regulators, osmoprotectants, and minerals is a feasible technique to cope with moisture deficit in the soil [13]. Foliar spraying of the potential growth regulator jasmonic acid has been found to enhance defense against reactive oxygen species (ROS) by regulating relative water content (RWC), photosynthetic rate, intercellular CO_2_ level, stomatal conductance, transpiration rate, and WUE [14]. Jasmonic acid boosts chlorophyll content by protecting photosystems and the electron transport chain [15], ultimately improving the overall growth of the root and shoot systems. Jasmonic acid is a potential plant growth regulator involved in metabolic pathways responsible for activating genes related to water stress management [16]. It enhances the rate of photosynthesis by improving chlorophyll-a and b content in soybeans, with a progressive effect on plant vigor after drought-induced injury [17]. Jasmonic acid mainly functions to improve plant tolerance against oxidative damage by enhancing the antioxidative role within organelles and maintaining homeostasis [18].

Sunflower is the most economical oil-seed crop, and its ability to thrive under water deficit conditions makes it an ideal climate-smart crop [19]. However, the average sunflower yield in Pakistan is lower than its potential, leading to a reliance on imported vegetable oil [20]. Therefore, there is a need to enhance sunflower production to ensure food security and meet the increasing demand of the growing population [21]. In contrast to other oil crops, sunflower is moderately drought-tolerant except during the flowering stage [22]. While foliar spray of jasmonic acid has shown promising effects in enhancing drought tolerance in other crops, its potential in sunflower cultivation is yet to be explored. By focusing on the development of drought-tolerant oil crops like sunflower, Pakistan can become self-sufficient in meeting its vegetable oil requirements.

This research work aimed to examine the jasmonic acid efficacy to alleviate the negative influence of drought stress on biomass production, physiological and biochemical aspects of potential seed oil sunflower crops.

## Materials and methods

### Plant material and treatments

This experimental trial was performed at the botanical garden of the University of Gujrat in the year 2022. Four varieties of sunflower (Armani gold, KQS-HSF-1, Parsun, and ESFH-3391) were acquired from the National Agriculture Research Center (NARC) Islamabad. Seeds sterilization was done with chlorine 5% and alcohol 95%, 10-seeds/pot of similar size were sown under usual day-light conditions in earthen pots having 12 kg of sandy loam soil (diameter of 21 cm, length 24.5 cm) with drainage holes at the bottom. There were 144 pots in the present experiment and 3 pots in each replicate. Before commencing the experiment, the physical and chemical properties of soil were determined using soil samples for laboratory analysis and results are shown in Table 1. The average day/night temperature was 36.11 ± 4/21.01 ± 3 ^o^C. Pots were arranged according to a completely randomized design. After two weeks of sowing, germination started, and the thinning was done to keep five healthy seedlings in each pot. All pots received an adequate water supply for two weeks after seedling emergence. Afterward, the drought stress treatments were initiated, controlled (0% drought stress), and treated (25%, 50%, and 75% drought stress). Moisture content was maintained on alternate days by maintaining the moisture level in the pots according to the specific field capacity in each treatment. Three levels of jasmonic acid, control (0ppm), 40ppm, and 80ppm were provided exogenously using a Style 1.5 compression sprinkler with nozzle size 120 micrometer (Matabi, Spain) as a foliar application in treated plants after 20 days of drought stress induction. In this experiment, German jasmonic acid (SIGMA-ALDRICH) was used with purity of more than 97%.

**Table 1:** Physical and chemical properties of soil

Soil properties	Measurement
Soil texture	Sandy loam
Sand (%)	65
Clay (%)	17
Silt (%)	18
pH	7.5
Electrical conductivity ds m^− 1^	2.13
Organic matter (%)	0.59

### Morphological traits and data collection

One plant from each replicate was selected and uprooted after fifteen days of foliar application of jasmonic acid to measure the following attributes i.e. shoot and root length and their biomass. The leaves in each treatment were picked and counted to record their number/plant. The above data were collected at the vegetative stage.

### Chlorophyll a and b content

To estimate the chlorophyll a and b content method [23] was used in leaves. For this investigation, a fresh leaf sample weighing 30 mg from each treatment was obtained. The homogenization of these samples was done in 3 ml of 80% acetone (v/v) and was centrifuged at 5000 rpm for 10 min to vanish all the particulate matter. By using the method of [24]the dilution of supernatant was done by adding acetone to make up 6 ml of the final volume. Chlorophyll a and b were calculated at 663 nm and 646 nm respectively by spectrophotometer using the following formulas.

$${\text{Chlorophylla}}\left( {\frac{{{\text{mg}}}}{{\text{g}}}} \right) = \frac{{\left( {12.7 \times {\text{A}}663} \right) - \left( {2.69 \times {\text{A}}645} \right) \times {\text{V}}}}{{1000 \times {\text{W}}}}$$

$${\text{Chlorophyllb}}\left( {\frac{{{\text{mg}}}}{{\text{g}}}} \right) = \frac{{\left( {22.9 \times {\text{A}}645} \right) - \left( {4.68 \times {\text{A}}645} \right) \times {\text{V}}}}{{1000 \times {\text{W}}}}$$

### Relative water content (RWC)

To determine the RWC, the third youngest leaf was picked, and 1 cm disks were removed from each sampled leaf. Five disks were chosen to immediately note the fresh biomass, later the disks were placed in distilled water for 24 h to get fully turgid weight (TW). Afterward, the disks were dried at 85 °C for 12 h in a hot air oven (Memmert, UNB 500, Germany) to get their dry weight (DW). According to the following formula [25]RWC was calculated.

$$RWC{\text{ }} = {\text{ }}FW{\text{ }}-{\text{ }}DW{\text{ }}/{\text{ }}TW{\text{ }}-{\text{ }}DW{\text{ }} \times {\text{ }}100$$

### Gas exchange characteristics

Data for gaseous exchange capacities was collected between 11:00 and 13:00 on clear sunny days. An expanded full-grown leaf per plant was selected from the aerial portion of a plant to measure gas exchange characteristics. Measurements of Stomatal conductance (gs) were done twice during the experiment. Photosynthesis rate (A) was determined with an LCA-4 ADC open-system portable infrared gas analyzer (ADC, Hoddesdon, England). The method of [26] was applied to calculate the transpiration rate, photosynthetic rate, and stomatal conductance. Following were the conditions within the portable meter chamber while taking readings: leaf temperature of 25 *±* 5 ^0^C, and leaf-to-air vapor pressure difference of 1.8 *±* 0.5 kPa. The total transpiration rate (E) was determined by the method of [27]. Plastic wrap was used to cover pots to avoid water loss and moisture content was noted twice a week to maintain specific field capacity and drought stress level.

Water Use Efficiency (WUE).

Data for WUE was calculated according to this formula.

$$WUE = \frac{{Photosynthetic\,rate\left( A \right)}}{{Transpiration\,rate\left( E \right)}}$$

### Malondialdehyde content

The membranous lipid peroxidation process under stress raised the production of malondialdehyde (MDA), its measurement was done with technique [28]. For this purpose, 300 mg of fresh leaf tissue was homogenously ground using a pestle and mortar and was stored in a solution of nitrogen. Afterward, centrifugation of these samples was done at 5000 rpm for 15 min, to estimate absorbance at 440 nm, 532 nm, and 600 nm for which a spectrophotometer (Hitachi- U2001, Tokyo, Japan) was used.

### H_2_O_2_ content

For hydrogen peroxide determination in sunflower leaves, the method of [29] was used. A fresh leaf tissue having 0.5 g weight was taken in an ice-cold pestle & mortar and ground with 5 ml of trichloroacetic acid thoroughly. The homogenous mixture was centrifuged for 15 min in 0.5 L aliquot, 1 ml of KI, and 0.5 ml buffering solution of KOH having neutral pH. The homogenous mixture was vortexed vigorously before determining the optical density at a wavelength of 390 nm using a spectrophotometer (Hitachi- U2001, Tokyo, Japan).

### Phenolic content

For determination of plant soluble phenolics a newly excised sunflower leaf sample was ground and homogenized in a previously ice-cold pestle and mortar with 80% acetone. Afterward, the homogenate was centrifuged for 10 min and allowed to settle down, then the supernatant was poured into a test tube. Deionized water 2 ml and Folin-Ciocalteau’s phenol reagent 1 ml were mixed with homogenate in the test tube. The additions were mixed thoroughly then added 5 ml (20%) Na_2_CO_3_, more water (distilled water) was poured into the reaction mixture to raise its volume to 10 ml. The contents were carefully mixed before measuring the optical density at 750 nm wavelength [30] using a spectrophotometer (Hitachi- U2001, Tokyo, Japan).

### Leaf proline content

To estimate the proline content in plant tissue, the method of [31] was used. For this purpose, a half-gram (0.5gm) of freshly taken leaf was homogenized using 3% sulfosalicylic acid (10 ml). Afterward, filtration of homogenate was carried out using filter paper (Whatman No. 2) and the volume was raised to 10 ml, 2 ml of ninhydrin reagent and 2 ml of filtrate, and glacial acetic acid (Sigma-Aldrich) was mixed thoroughly. Incubation of this mixture was done for an hour and boiled at 70^o^C by keeping it in a water bath, an ice bath was used to cool the reaction mixture. The extraction was done by vigorous shaking of the mixture with 4 ml of toluene for 15–20 s. A separating funnel was used for the separation of fractions of the reaction mixture. First, the top layer was removed, and the absorbance was noted at 520 nm using toluene as a control. A standard calibration curve is used to calculate the proline level. The instrument used for this parameter was a spectrophotometer (Hitachi- U2001, Tokyo, Japan).

### Glycine betaine

Samples of fresh leaf tissue weighing 0.5 g were finely ground in 10 ml distilled water and the contents were centrifuged. Then 1 ml supernatant, 1 ml of H_2_SO_4_ (2 N), and 0.2 ml KI_3_ were taken in a test tube, and the mixture in test tubes was kept for 1.5 h at 20^o^C in an ice bath for cooling. After that 6 ml of 1, 2 dichloroethane, and 2.8 ml of deionized water was added to the reaction mixture. Allowed to settle down, after 10 min two layers appeared in the test tube discarded the upper layer and the optical density was determined at 365 nm against the standard curve [32].

### Experimental design and statistical analysis

The experiment layout was completely randomized design with three replications. Data of all the attributes was analyzed using the Minitab statistical software and statistical significance was determined at a significance level of *p* ≤ 0.001.

## Results

### Morphological parameters

**Fig. 1:**
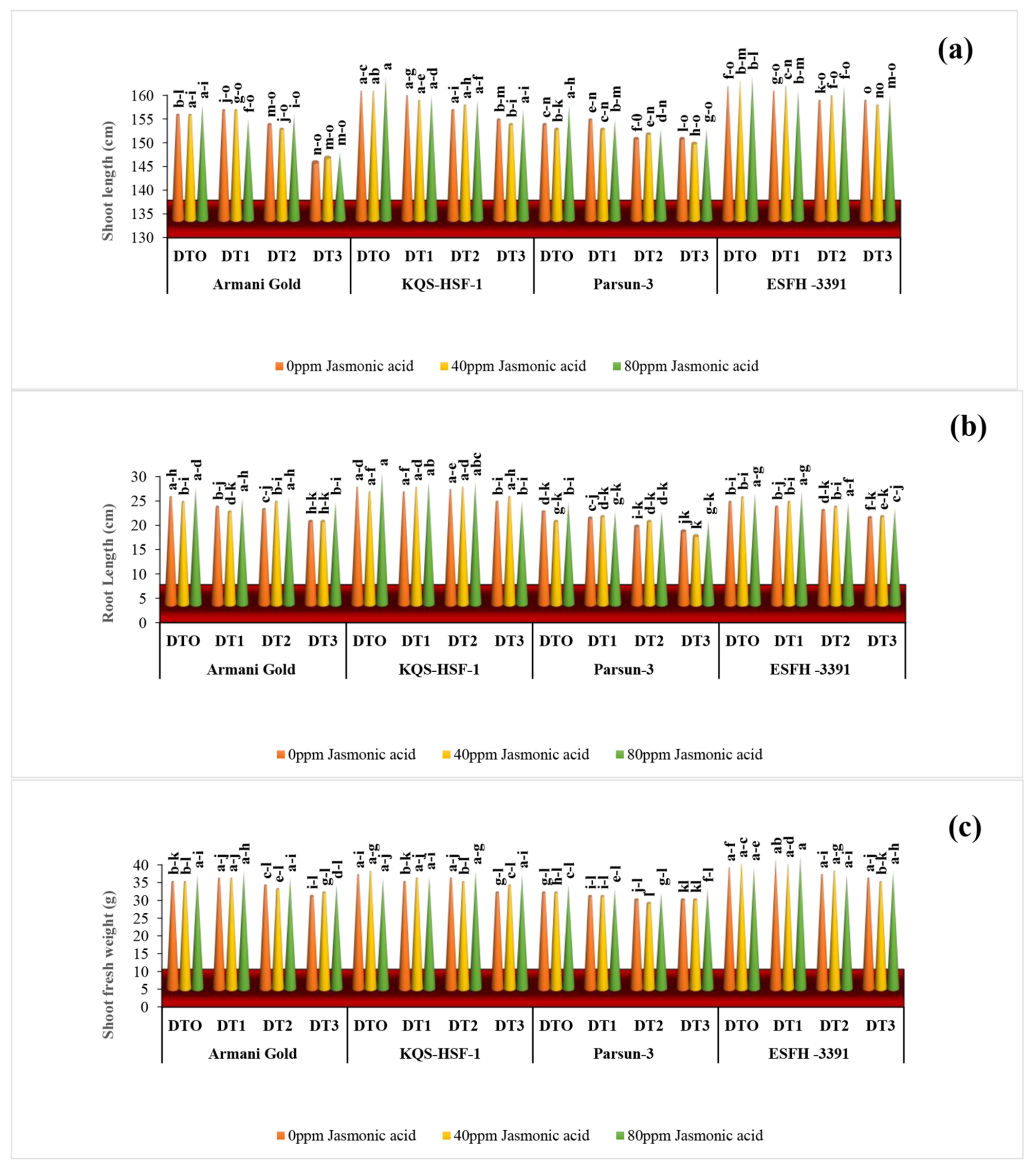
Shoot length** (a)**, root length **(b)** and shoot fresh weight **(c) **of four sunflower varieties treated with foliar-applied jasmonic acid under varying levels of drought stress. (DT0 = 0% drought stress, DT1 = 25% drought stress, DT2 = 50% drought stress, DT3 = 75% drought stress)

**Fig. 2:**
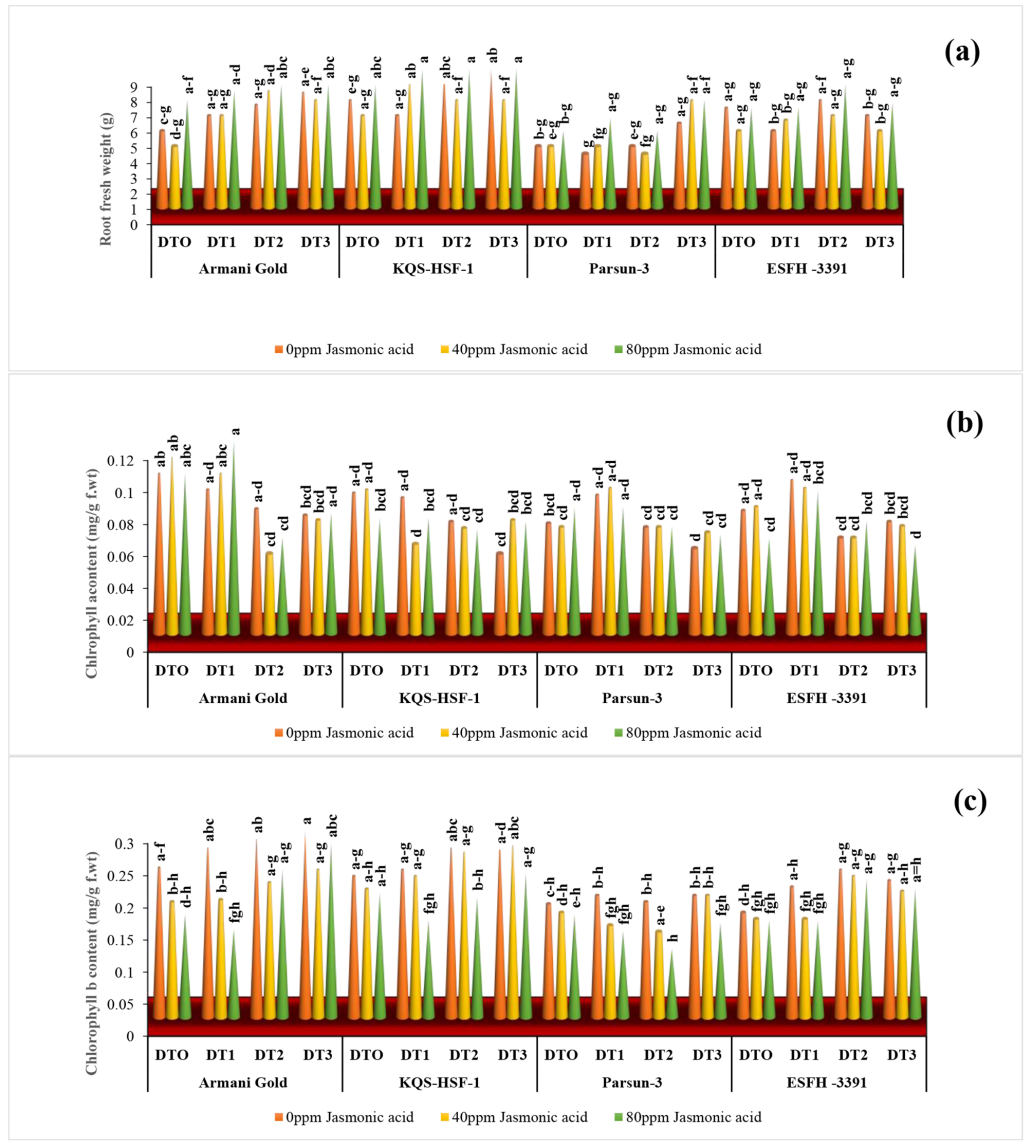
Root fresh weight **(a)**, chlorophyll a **(b)**, and chlorophyll b **(c)** of four sunflower varieties are treated to foliar-applied jasmonic acid under varying levels of drought stress. (DT0 = 0% drought stress, DT1 = 25% drought stress, DT2 = 50% drought stress, DT3 = 75% drought stress)

The length and fresh weight of the shoot and root of four sunflower varieties were significantly reduced due to drought stress. Severe drought stress (75%) caused a remarkable decrease in shoot and root length of Armani gold by 6.57% and 22.72% respectively. The exogenously applied 80ppm jasmonic acid significantly improved (*P* ≤ 0.001) these parameters in all sunflower varieties under both stressed and normal conditions with Armani gold showing an increase of 22%, KQS-HSF-1 of 12%, Parsun of 8%, and ESFH-3391 of 15%. Root length in Armani gold showed a 23% increase, and Parsun showed a 13% increase after the 80ppm JA application. Decrease in shoot fresh weight across all varieties: Armani Gold by 13%, KQS-HSF-1 by 15%, Parsun by 7%, and ESFH-3391 by 8%. Remarkably, KQS-HSF-1 exhibited the highest decrease in shoot fresh weight at 15% under severe drought stress (75%), followed by Armani Gold at 13%. The same trend was seen at the 75% drought stress level, where JA 80ppm effectively boosted shoot fresh weight for Armani Gold (11%), KQS-HSF-1 (18%), Parsun (12%), and ESFH-3391 (6%), in contrast to the limited effect observed at the same stress level with 40ppm JA. The impact of drought stress (75%) was evident in the reduction of root fresh weight across all sunflower varieties: Armani Gold decreased by 50%, KQS-HSF-1 by 57%, Parsun by 37%, and ESFH-3391 by 7%, respectively. ESFH-3391 demonstrated a consistent reduction of 7% under both 75% and 50% drought conditions. Among the varieties, Parsun exhibited the most substantial increase in root fresh weight, at 27%, under severe drought stress (75%) and with the application of 80ppm JA (Figs. 1 and 2).

### Biochemical parameters

#### Chlorophyll-a and b content

The increased drought stress level in the growth medium significantly reduced chlorophyll ‘a’ and ‘b’ in all sunflower varieties. However, values of chlorophyll ‘a’ were reduced in variety Armani gold, 68%, KQS-HSF-1, and Parsun 13%, ESFH-3391, 22% at 50% drought stress. While under severe drought stress (75%) reduction was more prominent. The foliar spray of 40ppm and 80ppm JA significantly improved the values of chlorophyll ‘a’ and b in sunflower hybrid varieties grown under water-stressed and control conditions correspondingly. However, under severe drought conditions, foliar applied 80ppm JA resulted in optimum improvement in chlorophyll ‘a’ in Parsun, 40% whereas, strangely Armani gold expressed 1.4% improvement when applied 80ppm JA. Under severe drought (75%) Parsun was more drought tolerant with less change in chlorophyll ‘b’. The higher dose of 80ppm JA exogenous spray resulted in a more significant (*p* < 0.001) change as, Armani gold by 15%, KQS-HSF-1 by 27%, Parsun, 39% with the highest percentage was more drought tolerant under this stress level. Both JA levels of 40ppm and 80ppm under severe drought stress (75%) expressed less improvement as compared to moderate drought stress (50%) effect alleviation (Fig. 2).

### Leaf proline and glycine betaine content

**Fig. 3:**
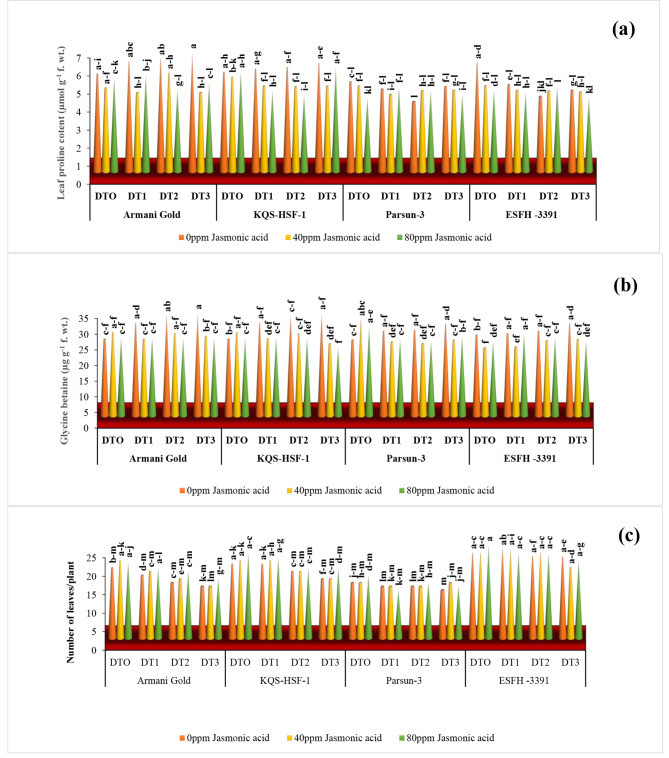
Leaf proline **(a)**, leaf glycine beatine content **(b)** and number of leaf/plants **(c)** of four sunflower varieties treated with foliar-applied jasmonic acid under varying levels of drought stress. (DT0 = 0% drought stress, DT1 = 25% drought stress, DT2 = 50% drought stress, DT3 = 75% drought stress)

Proline content increased with increasing levels of drought stress conditions, in variety Armani gold proline level increased by 22%, and in KQS-HSF-1 it was 9% as compared to the control group. Contrarily variety Parsun and ESFH-3391 showed a reduction in proline level by 6% and 25% under severe drought stress (75%). Armani gold was the most tolerant among all by a maximum increase in proline level under severe drought stress. In the case of moderate drought stress (50%), Armani gold and KQS-HSF-1 exhibited a rise in proline level of 15% and 5% respectively, and in Parsun, 23% and in ESFH-3391 by 31% unusually. Both JA levels reduced proline content in all varieties i.e., in Armani gold 33% was the maximum value followed by 21% in KQS-HSF-1 under 75% water stress and 40ppm JA treatment. While under severe drought 80ppm JA effect was less than 40ppm spray. Though, the maximum increase in GB contents was noted in Armani gold and KQS-HSF-1 by 35% as compared to control under severe water stress (75%) conditions, in Parsun and ESFH-3391 it remained by 29% under the same stress level. 80ppm JA application under moderate stress level decreased GB by 23% in Armani gold and KQS-HSF-1, 11% in Parsun, and 7.5% in ESFH-3391 (Fig. 3).

### Number of leaves/plants

The imposition of water deficiency significantly (*P* ≤ 0.001) suppressed the number of leaves per plant across all sunflower varieties under varying levels of drought stress (50%, 75%) in the growth medium. Under severe drought stress (75%), Armani Gold exhibited the most substantial reduction in leaves per plant at 26%, followed by KQS-HSF-3391 at 20%, in comparison to the control group (Fig. 3). The application of exogenous jasmonic acid at 80ppm significantly improved the number of leaves of sunflower varieties (*P* ≤ 0.01), and the effect on variety response to jasmonic acid was also non-significant (*P* ≥ 0.01).

### Total phenolic content

**Fig. 4:**
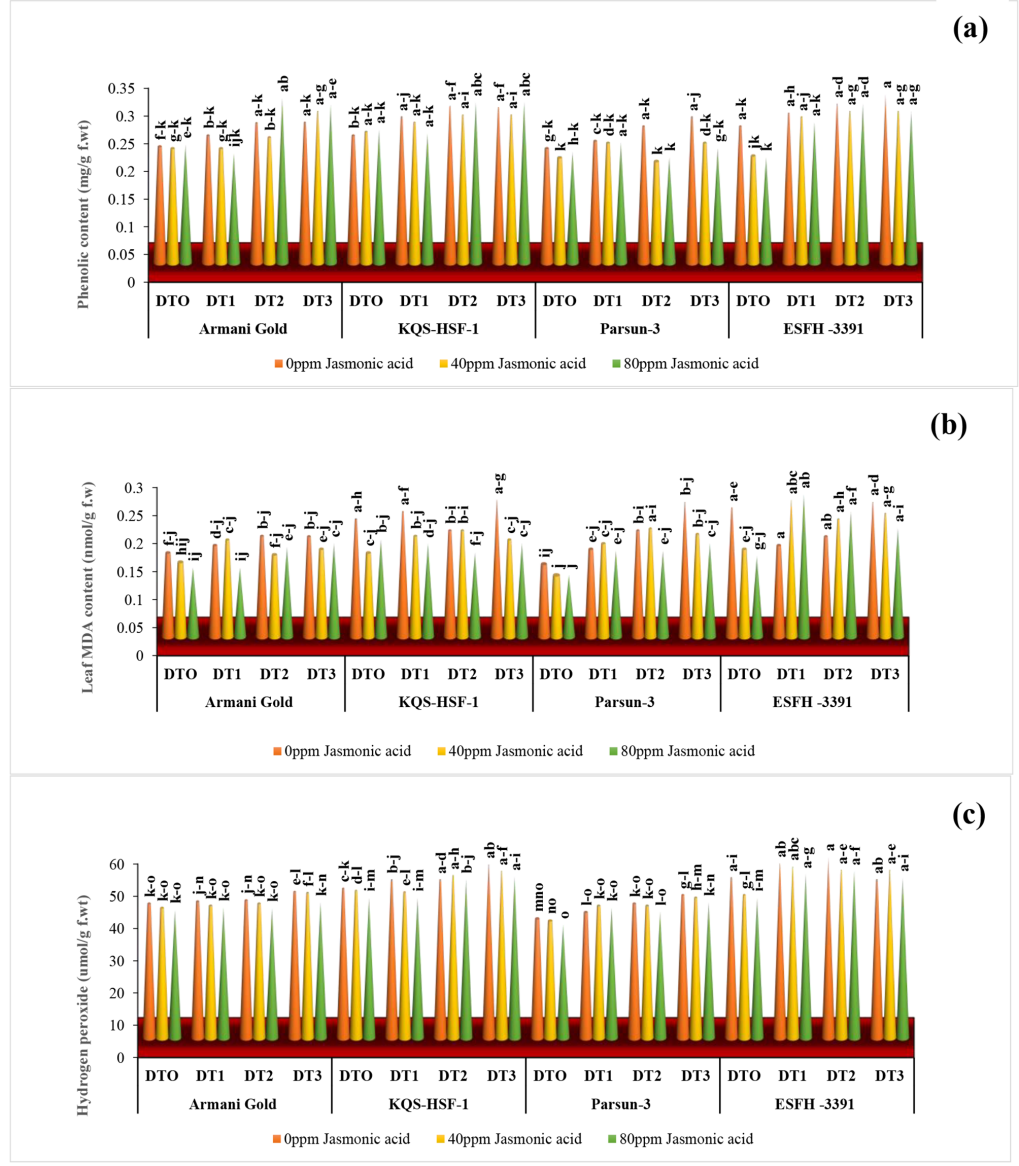
Phenolic contents **(a)**, leaf MDA content **(b)** and hydrogen peroxide **(c) **of four sunflower varieties treated with foliar-applied jasmonic acid under varying levels of drought stress. (DT0 = 0% drought stress, DT1 = 25% drought stress, DT2 = 50% drought stress, DT3 = 75% drought stress)

Total phenolic content in sunflower varieties improved (*P* ≤ 0.001) significantly under drought stress conditions (Fig. 4). Under stress conditions (75%), variety Parsun, 23% showed the highest phenolic contents, and variety Armani gold, 19% showed minimum accumulation of total phenolics under varying water regimes (50%, 75%). It was noticed after 40ppm and 80ppm JA exogenous application the phenolics reduced minutely in all varieties, except Parsun with a maximum of 27% and 28% rise under both levels of JA and 50% drought stress.

### Malondialdehyde (MDA) content

Water stress regime promoted (*P* ≤ 0.001) malondialdehyde levels in all sunflower plants. Moreover, Jasmonic acid as foliar treatment helped lower MDA content in all experimental and control sunflower varieties significantly. There was a uniform response of all varieties under severe drought stress (75%) for this parameter except Armani gold with 20%, KQS-HSF-1, 14%, Parsun, 85%, and ESFH-3391, 4.5% as compared to the control group. For foliar spray of 80ppm JA, the percentage was higher: Armani gold by 11%, KQS-HSF-1, and parsun by 21%, and ESFH-3391 by 22%. Among the two concentrations (40 ppm and 80 ppm) of Jasmonic acid, 80 ppm foliar spray notably decreased MDA compared to the control under moderate and severe drought stress levels (50% and 75%) (Fig. 4).

### Hydrogen peroxide (H_2_O_2_)

It was observed that hydrogen peroxide contents in all varieties significantly (*P* ≤ 0.001) (Fig. 4) enhanced underwater deficit conditions except Armani gold. The maximum increase was experienced by Parsun i.e. 19% under severe drought stress (75%), KQS-HSF-1 was the second-best variety with a 17% rise in hydrogen peroxide value. Similarly, under moderate drought stress (50%) Parsun and KQS-HSF-1 possessed 14% and 12% more H_2_O_2_ as compared to control. Jasmonic acid 40ppm and 80ppm caused a little reduction (*P* ≤ 0.001) in hydrogen peroxide value showing a significant effect.

### Gas exchange characteristics

The gas exchange characteristics values, namely the photosynthetic rate, transpiration rate, stomatal conductance, and sub-stomatal CO_2_ concentration, in all sunflower varieties, experienced reduction in a water-deficient environment. The reductions in photosynthetic rate were 17% for Armani gold, 10% for KQS-HSF-1, and 9% for Parsun at 75% drought stress, indicating their limited resistance against drought. With 80ppm JA, Armani gold saw a substantial increase of 22%, KQS-HSF-1 by 14%, and ESFH-3391 by 12% at 75% drought stress. Under 50% drought stress, the application of 40ppm JA resulted in a similar level of increase as observed under severe drought, while 80ppm JA led to a net increase of 16% in Armani gold, followed by ESFH-3391 with 14%, and 6–7% in KQS-HSF-1 and Parsun, respectively.

Severe drought stress significantly reduced the transpiration rate by 40% in Armani gold and KQS-HSF-1, while Parsun exhibited a 50% reduction compared to ESFH-3391’s 20% reduction. ESFH-3391 consistently expressed the least reduction and maintained a high tolerance to drought stress. At 80ppm JA, the transpiration rate increased by 20% in Armani gold and 40% in Parsun. When 40ppm JA was applied under severe drought (75%), Armani gold showed a 25% increase, and Parsun exhibited a 50% increase in transpiration rate. Under 80ppm JA, these increases were 33% in Armani gold and 50% in Parsun.

**Fig. 5:**
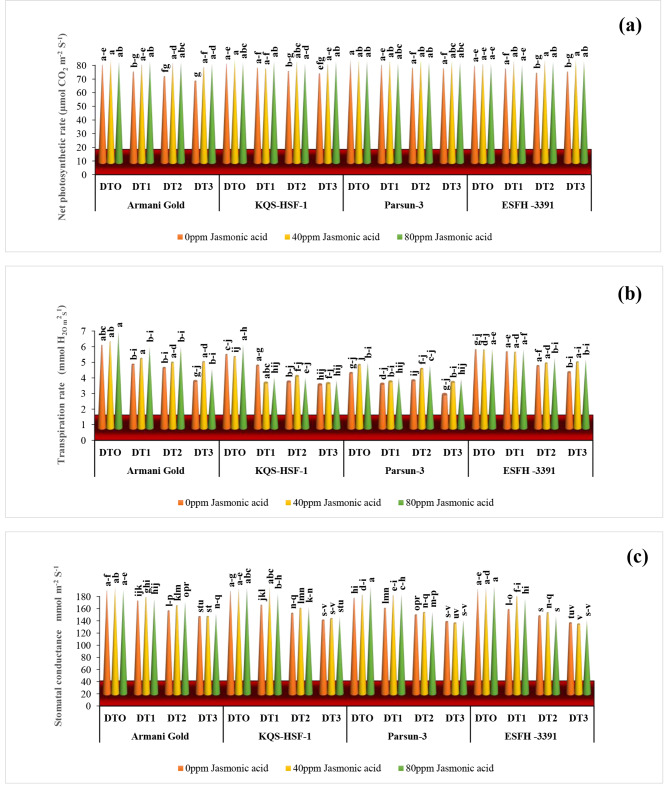
Net photosynthetic rate **(a)**, transpiration rate **(b)** and stomatal conductance **(c)** of four sunflower varieties treated with foliar-applied jasmonic acid under varying levels of drought stress. (DT0 = 0% drought stress, DT1 = 25% drought stress, DT2 = 50% drought stress, DT3 = 75% drought stress)

Under severe drought stress (75%), stomatal conductance was reduced in all varieties except for KQS-HSF-1, which exhibited a 28% reduction and was the most sensitive to drought. In the case of moderate drought stress (50%), the reductions were slightly different than under severe drought: Armani gold reduced by 20%, KQS-HSF-1 by 21%, Parsun by 18%, and ESFH-3391 by 26%. Both concentrations of JA, 40 ppm and 80 ppm, were equally effective in improving stomatal conductance under moderate drought stress (50%), except for Armani gold, which exhibited the maximum improvement of 12% at 80 ppm JA. The sub-stomatal CO_2_ concentration showed significant (*P* < 0.001) reductions in drought-stressed plants of all sunflower varieties compared to non-stressed plants (Fig. 5).

**Fig. 6:**
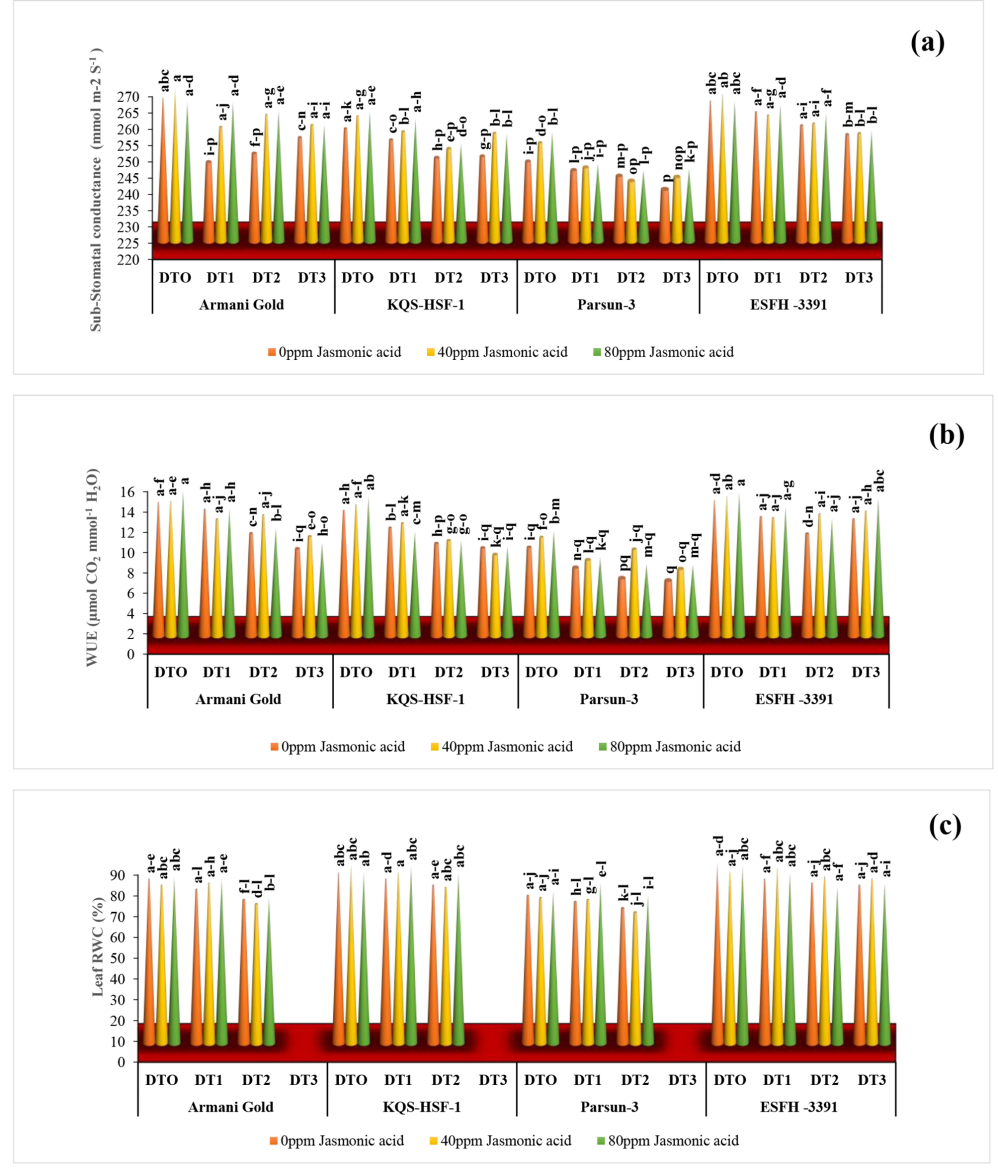
Sub-stomatal CO_2_ conc. **(a)**, water use efficiency (WUE) **(b)** and leaf relative water content **(c) **of four sunflower varieties treated with foliar-applied jasmonic acid under varying levels of drought stress. (DT0 = 0% drought stress, DT1 = 25% drought stress, DT2 = 50% drought stress, DT3 = 75% drought stress)

Under severe drought stress (75%), the leaf sub-stomatal CO_2_ concentration was equally decreased across all varieties within a narrow range of 3–5% compared to normal conditions. The foliar-applied jasmonic acid at the level of 80ppm effectively overcame the effect of drought stress and enhanced the values of sub-stomatal CO_2_ concentration (Fig. 6).

### Water use efficiency

Drought stress had a significant (*P* < 0.001) impact on the water use efficiency (WUE) of all sunflower varieties. Under severe drought stress (75%), Armani gold, KQS-HSF-1, Parsun, and ESFH-3391 exhibited WUE values of 31%, 25%, 33%, and 8% respectively, which were lower than those in control plants. Similarly, under moderate drought stress (50%), Armani gold and ESFH-3391 experienced a 23% reduction in WUE compared to KQS-HSF-1 (25%) and Parsun (33%). Notably, ESFH-3391 demonstrated the status of a drought-tolerant variety with only an 8% reduction in WUE under severe drought stress (75%). Foliar application of different levels of JA significantly (*P* < 0.001) enhanced the water use efficiency of all varieties (Fig. 6). In general, the foliar application of JA notably increased WUE in a variety of ESFH-3391 and Parsun.

### Relative water content

The leaves’ relative water content (RWC) of sunflowers significantly decreased due to increasing levels of drought stress in the growth medium (*p* < 0.001). Severe drought stress (75%) caused a remarkable reduction in the RWC of all varieties: Armani gold by 10%, KQS-HSF-1 by 4%, Parsun by 16%, and ESFH-3391 by 4.6%. Among all varieties, KQS-HSF-1 demonstrated the least reduction in RWC under severe drought stress (75%). Notably, the application of an exogenous spray of Jasmonic Acid (80ppm) under moderate drought stress (50%) and control conditions resulted in increased RWC for all varieties, with Armani gold showing an increase of 6.6%, KQS-HSF-1 and Parsun at 3%, and ESFH-3391 at 2.6%. Foliar application of 80ppm JA during drought stress (75%) also enhanced RWC, leading to the following improvements: Armani gold at 6%, KQS-HSF-1 at 4%, Parsun at 2.7%, and ESFH-3391 at 0.80% (Fig. 6).

## Discussion

Exposure of plants to drought stress stimulates the synthesis of osmoprotectents [33] in lower concentrations, making it hard for plants to withstand challenging situations [34]. To maintain optimal endogenous levels, growth regulators can be applied exogenously [35]. Jasmonic acid (JA), a crucial plant growth regulator, plays a pivotal role in combating drought stress; it plays a fundamental role in stabilizing dehydrated enzymes and destruction of cellular membranes [36].

This study utilized the exogenous application of JA to assess its effectiveness and suitable dose for enhancing the antioxidant capability of sunflower against drought stress. Encouragingly, plant growth improved after the application of JA (40 ppm and 80 ppm) under varying levels of drought stress. Drought significantly suppressed the shoot and root length of all sunflower varieties, because of restricted translocation from leaves, ultimately disrupting mitosis in the shoot and root meristem. Generally, low moisture content enables the root system to grow faster than the shoot, indicating that roots are comparatively less vulnerable to water stress [37]. These findings are consistent with those of [14], who hypothesized that under drought stress, foliar spray of jasmonic acid enhanced the root and shoot length of radish. Drought-induced growth patterns in the root help to cope with water stress, as it represents an emergency strategy adopted by root cells [38]. These outcomes can be associated with previous studies where the foliar spray of jasmonic acid was applied to crops under stressful conditions, such as black nightshade [39], maize [40], soybean [41], radish [14], tomato [39]). The number of leaves reduced in all sunflower varieties after enduring drought stress. Water stress leads to premature leaf fall and senescence, reducing the number of leaves per plant in dry beans [42–47]. Furthermore, JA application increased the number of leaves per plant by maintaining leaf turgor pressure and enhancing gas exchange attributes. Similar conclusions were drawn by [18]in their research on crops.

Osmoregulation between mesophyll tissues and transpiration is closely linked to relative water content (RWC), which serves as a fundamental indicator of a plant’s ability to cope with low soil moisture levels [48]. A reduction in RWC is associated with decreased water transport via the xylem to the leaf cells due to reduced transpiration through stomata [49–53]. Water stress in the growth medium reduced turgor pressure at the cellular level, resulting in a decline in RWC in mesophyll cells and guard cells [54]. The application of jasmonic acid enhanced the water status of sunflower plants by boosting defensive biochemical activities and secondary metabolites to improve resilience. Similar findings were reported in radish [55, 56], sugar beet [57] and cotton [58]. In this study, a significant increase in RWC was observed at a higher dose of JA 80ppm under drought stress.

Upon the onset of drought stress, stomatal conductance is reduced, due to turgor loss in guard cells. As a result, the photosynthetic rate is affected because of protein structural instability that connects photosystem I and II in safflower and lemon grass [59]. The reduced availability of carbon dioxide to Ribulose-1, 5-bisphosphate (RuBP) is also responsible for the reduced photosynthetic rate [42]. Jasmonic acid treatment improved the rate of photosynthesis in all varieties, but a non-significant effect of JA was noted in two varieties, which aligns with the findings of [36, 60] found that jasmonic acid application enhanced the rate of photosynthesis as well as the rate of transpiration in Brassica species under both water deficits. The transpiration rate was decreased by moisture stress in all sunflower varieties under severe conditions. A similar trend was observed in tomatoes [39, 61] where exogenous application of JA improved physiological activities and stomatal conductance. Under drought stress, sub-stomatal CO_2_ concentration decreased in all sunflower plants, primarily due to the closure of stomata, which led to further reduction in photosynthesis [62]. Conversely, JA foliar spray enhanced sub-stomatal carbon dioxide levels by bolstering the plant’s defense system with antioxidants and ROS scavengers. JA application significantly improved this vital attribute by playing a protective role against water stress and harmful metabolites in plants [5], similar findings were observed in strawberries [63].

Water deficit conditions elevate the ROS levels and cause a reduction in the rate of photosynthesis due to damage to chlorophyll a, and b molecules in the thylakoid membrane and photosystems [33], ultimately leading to a complete loss of pigments [64]. Similar results were observed in the present study under moderate (50%) and severe (75%) drought conditions, which significantly reduced photosynthetic pigments. Jasmonic acid, applied via foliar application, equally enhanced chlorophyll content in all sunflower varieties under both stressed and non-stressed conditions. It was observed that priming radish seeds with JA led to a significant increase in chlorophyll-a content, attributed to its protective role in photosystems and enzyme synthesis [14]. In the present study, a decrease in chlorophyll-b content was observed under water-deficient conditions. However, the results of [65]in German chamomile (*Matricariac chamomilla* L.) and [66] in basil plants (*Ocimum basilicum* L.) contradicted our findings as they observed a reduction in chlorophyll-b pigment levels under drought stress.

Proline and glycine betaine are among the most active and major osmolytes that play a crucial role in tolerating various abiotic stresses. In this research, all drought treatments showed a significant increase in both glycine betaine (GB) and proline concentrations in all sunflower varieties. However, plants provided with JA exhibited a reduction in proline and GB activity under drought stress. These results contrast with the observations [36], those who reported better accumulation of proline in seedlings of wheat and Brassica when JA was applied under water deficit conditions. In a study conducted by [67–69], exogenous application of JA and pre-sowing seed treatment with JA resulted in elevated levels of proline in drought-stressed plants of poplar (*Populus alba* L.) and honey flower (*Melianthus comosus* L.). Additionally, JA may induce the expression of proline synthesis genes to promote a higher proline level. These findings are consistent with previously investigated data on sunflower [45] as underwater shortage GB modifies biochemical attributes of osmotic adjustment at the cellular level.

Plants accumulate phenolic contents to counter the harmful effects of oxidative agents during stressful environments [70]. In our experiments, dehydration stress increased the total phenolic contents in all sunflower varieties. This aligns with previous reports [71], where the phenolics contents were in peppermint.

In the current study, it was observed that the concentration of hydrogen peroxide increased in all sunflower varieties when exposed to various drought stress levels. However, the exogenous application of jasmonic acid had a significant impact on H_2_O_2_ by decreasing its level in all treatment groups. Similar findings were reported by other researchers, such as in sugar beet [47]. Furthermore, JA treatment reduced membrane lipid peroxidation, resulting in the accumulation of hydrogen peroxide in pearl millet and enhancing drought tolerance [14].

An increase in leaf MDA content was observed in all sunflower varieties, reflecting similar findings in wheat plants [72]. This increase is attributed to drought-induced membrane lipid peroxidation and elevated ROS levels. In our experiment, the external application of JA significantly reduced MDA levels in plants subjected to dehydration stress. Several researchers have reported a beneficial role of JA in reducing MDA contents, as seen in wheat [73], sugar beet [47], and cotton [74]. However, in contrast [48], found no influence of JA spray on cotton plants, possibly due to the high buildup of MDA contents under drought stress. Exogenous sprays of jasmonic acid caused lower membrane lipid peroxidation since this molecule favors the antioxidant system and improves the structure and stability of membranes.

The agricultural sector is currently facing multiple challenges that have negative effects on crop yield and quality [75]. In many developing countries, sunflower production faces significant limitations, including the lack of hybrid germplasm, severe weather conditions, inconsistent rainfall, market price instabilities, expensive farm inputs, disease, and bird attacks, a lack of modern mechanized farming, grower ignorance, and tough competition in the global oil importation market. Some farmers often continue cultivation on the same land, leading to soil overexploitation and common problems such as leaf spotting and head rots. Solutions to these problems include crop rotation, advanced irrigation technology, and the use of bio-pesticides [22]. Sunflower attracts many vertebrate and invertebrate pests, causing significant losses in the field. Proper measures are needed to address this issue [76].

Diseases and pest attacks during sunflower production, processing, and storage are significant challenges for this crop [52]. Sunflower requires less water compared to other plants due to their long and extensive taproot, which efficiently absorbs water from available soil moisture during drought conditions. A lack of information about sunflower farming techniques, poor processing, and marketing are significant challenges for uninformed stakeholders. Planting hybrid cultivars and practicing good agricultural techniques are ways to achieve optimum sunflower yields. Cultivating sunflowers for more than three successive seasons on the same farmland can lead to disease build-up. To prevent airborne transmission of sunflower diseases, farmers should use sanitized seeds. Prolonged monoculture in any soil leads to nutrient depletion. To address this issue, intercropping with multiple crops can result in better yield output [77].

## Conclusion

This research work was conducted to verify whether exogenously applied jasmonic acid (JA) plays a significant role in mitigating the deleterious consequences of drought stress on the morphological, physiological, and biochemical parameters of four sunflower varieties. Drought stress reduced the plant height, biomass, and number of leaves by up to 50% but the application of JA significantly improved water stress tolerance by improving drought effects by up to 10%. Foliar application of JA was equally effective in mitigating the adverse effects of drought stress on biochemical attributes like chlorophyll content, hydrogen peroxide, phenolics, MDA, proline, and glycine betaine, and gas exchange parameters enhanced (15–20%). The 80ppm JA concentration was more effective as compared to 40ppm in biomass production and chlorophyll content enhancement. Exogenous JA application improved photosynthesis in water-stressed sunflower plants by 15% due to improvement in stomatal conductance, RWC and improved WUE up to 17%. Results indicate that the application of 80ppm jasmonic acid more effectively mitigated drought stress, resulting in a 50–75% improvement in biomass production, biochemical attributes, and photosynthetic rate.

## Data Availability

The author confirms that all data generated or analyzed during this study are included in this published article.

## Declarations

### Study protocol must comply with relevant institutional, national, and international guidelines and legislation

Our experiment follows the relevant institutional, national, and international guidelines and legislation.
